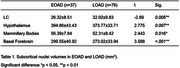# Sleep Disruption in Early‐Onset Alzheimer's Disease

**DOI:** 10.1002/alz70857_099055

**Published:** 2025-12-24

**Authors:** Neus Falgàs Martínez, Andrea Val‐Guardiola, Marta Peña, Gerard Mayà, Agnés Pérez‐Millan, Bea Bosch, Guadalupe Fernandez‐Villullas, Mircea Balasa, Carles Gaig, Adrià Tort‐Merino, Albert Lladó, Emma Muñoz‐Moreno, Álex Iranzo, Lea T. Grinberg, Raquel Sánchez‐Valle

**Affiliations:** ^1^ Neurology Service, Hospital Clínic de Barcelona and Institut D'Investigacions Biomèdiques August Pi i Sunyer (IDIBAPS), Barcelona, Spain; ^2^ Alzheimer's disease and other cognitive disorders unit, Hospital Clínic, IDIBAPS, Barcelona, Spain; ^3^ IDIBAPS, Barcelona, Barcelona, Spain; ^4^ Alzheimer's disease and other cognitive disorders Unit. Hospital Clínic. Fundació Clínic per a la Recerca Biomèdica, IDIBAPS, Universitat de Barcelona, Barcelona, Spain; ^5^ Alzheimer's disease and other cognitive disorders Unit. Hospital Clínic de Barcelona. Fundació de Recerca Clínic Barcelona – IDIBAPS. University of Barcelona, Barcelona, Spain; ^6^ Alzheimer's Disease and Other Cognitive Disorders Unit, Neurology Department, Hospital Clinic, Barcelona, Spain; ^7^ Image Diagnostic Centre, IDIBAPS, Hospital Clínic de Barcelona, Barcelona, Spain, Barcelona, Spain; ^8^ Memory and Aging Center, UCSF Weill Institute for Neurosciences, University of California, San Francisco, San Francisco, CA, USA; ^9^ Alzheimer's disease and other cognitive disorders Group. Service of Neurology, Hospital Clínic de Barcelona. Fundació Recerca Clínic Barcelona‐IDIBAPS, Barcelona, Spain

## Abstract

**Background:**

Atypical variants of sporadic Alzheimer's disease (AD) are characterized by earlier onset (EOAD, before age 65), non‐amnestic cognitive patterns, and distinctive neuropsychiatric and sleep‐related traits. Earlier onset has been linked to greater severity of neuropsychiatric symptoms and increased use of antidepressants or sleep medications. Furthermore, atypical variants such as Posterior Cortical Atrophy and the logopenic variant of Primary Progressive Aphasia exhibit pronounced REM sleep dysfunction. The mechanisms driving these behavioral and sleep differences, influenced by age at onset and atypical disease features, remain unclear. Emerging evidence suggests that a differential pattern of degeneration within the neuromodulatory subcortical systems (NSS), including the locus coeruleus (LC), may contribute to these distinct profiles. This study compared sleep‐wake patterns and volumes of NSS in EOAD and late‐onset AD (LOAD).

**Method:**

The study included 113 biomarker‐confirmed AD participants (37 EOAD, 76 LOAD) in the early stages (mild cognitive impairment or mild dementia). All participants underwent MRI with quantification of subcortical nucleus volumes. A subset of 66 participants (23 EOAD, 43 LOAD) completed the Pittsburgh Sleep Quality Index (PSQI), Epworth Sleepiness Scale (ESS), and two weeks of actigraphy monitoring using the Motion Watch8 device. Sleep‐wake patterns and circadian rhythms were analyzed using MotionWare software.

**Result:**

EOAD and LOAD participants had similar functional status (GDS3: 57% vs 55%), neuropsychiatric medication use (46% vs 42%), and subjective sleep assessments (PSQI: 6.55±4.33 vs 6.98±3.89; ESS: 6.45±4.35 vs 5.48±4.01). However, actigraphy revealed shorter total sleep time (384.07±67.06 vs. 425.00±58.09 min, *p* < 0.05) and more fragmented, unstable sleep patterns (384.13±68.57 vs. 419.33±57.17 min, *p* < 0.05) in EOAD compared to LOAD. Unlike other subcortical nuclei, LC volume was significantly smaller in EOAD than in LOAD (26.32±8.51 vs. 32.02±9.83 mm^3^) (see Table 1).

**Conclusions:**

While subjective sleep measures were similar between EOAD and LOAD, actigraphy revealed more significant sleep disruption in EOAD. Notably, LC volume was uniquely reduced in EOAD, whereas other subcortical nuclei showed larger volumes than LOAD. These findings highlight the need to explore the role of subcortical degeneration in sleep disturbances in early‐onset AD.